# Impact of Extended-Duration Shifts on Medical Errors, Adverse Events, and Attentional Failures

**DOI:** 10.1371/journal.pmed.0030487

**Published:** 2006-12-12

**Authors:** Laura K Barger, Najib T Ayas, Brian E Cade, John W Cronin, Bernard Rosner, Frank E Speizer, Charles A Czeisler

**Affiliations:** 1 Division of Sleep Medicine, Department of Medicine, Brigham and Women's Hospital, Boston, Massachusetts, United States of America; 2 Division of Sleep Medicine, Harvard Medical School, Boston, Massachusetts, United States of America; 3 Sleep Disorders Program, University of British Columbia, Vancouver, British Columbia, Canada; 4 Department of Medicine, Vancouver General Hospital, Vancouver, British Columbia, Canada; 5 Center for Clinical Epidemiology and Evaluation, Vancouver Coastal Health Research Institute, Vancouver, British Columbia, Canada; 6 Channing Laboratory, Department of Medicine, Brigham and Women's Hospital, Harvard Medical School, Massachusetts, United States of America; Stanford University, United States of America

## Abstract

**Background:**

A recent randomized controlled trial in critical-care units revealed that the elimination of extended-duration work shifts (≥24 h) reduces the rates of significant medical errors and polysomnographically recorded attentional failures. This raised the concern that the extended-duration shifts commonly worked by interns may contribute to the risk of medical errors being made, and perhaps to the risk of adverse events more generally. Our current study assessed whether extended-duration shifts worked by interns are associated with significant medical errors, adverse events, and attentional failures in a diverse population of interns across the United States.

**Methods and Findings:**

We conducted a Web-based survey, across the United States, in which 2,737 residents in their first postgraduate year (interns) completed 17,003 monthly reports. The association between the number of extended-duration shifts worked in the month and the reporting of significant medical errors, preventable adverse events, and attentional failures was assessed using a case-crossover analysis in which each intern acted as his/her own control. Compared to months in which no extended-duration shifts were worked, during months in which between one and four extended-duration shifts and five or more extended-duration shifts were worked, the odds ratios of reporting at least one fatigue-related significant medical error were 3.5 (95% confidence interval [CI], 3.3–3.7) and 7.5 (95% CI, 7.2–7.8), respectively. The respective odds ratios for fatigue-related preventable adverse events, 8.7 (95% CI, 3.4–22) and 7.0 (95% CI, 4.3–11), were also increased. Interns working five or more extended-duration shifts per month reported more attentional failures during lectures, rounds, and clinical activities, including surgery and reported 300% more fatigue-related preventable adverse events resulting in a fatality.

**Conclusions:**

In our survey, extended-duration work shifts were associated with an increased risk of significant medical errors, adverse events, and attentional failures in interns across the United States. These results have important public policy implications for postgraduate medical education.

## Abstract 2

During months in which medical interns worked extended shifts, the chances of their reporting at least one fatigue-related significant medical error increased more than 3-fold compared to months with no extended shifts.

## Editors' Summary

**Background.:**

In the United States, medical students who are doing their internship (first year of postgraduate clinical training) regularly work in the clinic for longer than 24 h at a time. It is already known that doctors or students who work for long shifts make more medical errors and are less able to pay attention to what they are doing. Many thousands of adverse medical events per year including, in the extreme, deaths of patients, are thought to result from medical errors, but it is not clear whether doctors or students working long shifts—as opposed to, for example, an increase in total number of hours worked—are the cause of many or any of these errors.

**Why Was This Study Done?:**

This research group wanted to find out whether long shifts worked by interns had an effect on reported medical errors, and hence patient safety, and specifically whether any harm that happened to patients might otherwise have been preventable.

**What Did the Researchers Do and Find?:**

The researchers contacted all US medical school graduates beginning their internships from one particular year-group by email, and asked each person whether they wanted to take part in a confidential survey. Individuals who agreed to participate were directed to a secure website to enter basic information about themselves and then to complete a form each month. On that form the interns gave information about their working hours, hours of sleep, and number of extended-duration shifts worked, and completed questions about medical errors in the past month. Then, for each intern in the study, researchers compared month by month the number of medical errors and the number of extended-duration shifts that had been worked. A total of 2,737 interns took part in the survey.

Compared to months in which no extended-duration shifts were worked, in those months in which between one and four, and more than five extended-duration shifts were worked, the doctors were, respectively, three and seven times more likely to report at least one fatigue-related significant medical error. Similarly, fatigue-related adverse events increased by around seven and eight times, respectively, compared with months in which no extended-duration shifts were worked. Fatigue-related preventable adverse events associated with the death of the patient increased by ∼300% in interns working more than five extended-duration shifts per month; they were also more likely to fall asleep during lectures, rounds, and clinical activities, including surgery.

**What Do These Findings Mean?:**

Guidelines for graduate medical education in the United States still allow up to nine marathon shifts (30 h at a stretch) per month, even though the total number of hours worked is capped. This study shows that the long shifts worked by interns are bad for patient safety, as they are more likely to cause harm that would not otherwise happen.

**Additional Information.:**

Please access these Web sites via the online version of this summary at http://dx.doi.org/doi:10.1371/journal.pmed.0030487.

- The US Food and Drug Administration has resources on its website about medication errors
- The National Sleep Foundation aims to improve public health and safety by achieving understanding of sleep and sleep disorders
- Wikipedia (an internet encyclopedia anyone can edit) has a page about residency training in the United States

## Introduction

Although the Accreditation Council for Graduate Medical Education (ACGME) has recently placed limitations on resident work hours in an attempt to reduce fatigue-related medical errors, the practice of working for more than 24 h consecutively remains the cornerstone of American postgraduate medical education. Moreover, a 1999 report from the Institute of Medicine revealed that between 48,000 and 98,000 deaths each year occur due to a medical error [1].

A recent randomized trial reported that interns working extended-duration shifts (defined as at least 24 h continuously at work) had significantly more polysomnographically recorded attentional failures and made significantly more serious medical errors than those scheduled to work shifts 16 h or longer [2,3]. However, that trial was not large enough to determine whether extended-duration work shifts increased the risk of preventable adverse events, for example injury due to a non-intercepted serious error in medical management, although a trend in that direction was reported [3]. To address the impact of extended-duration work shifts on patient safety in a large and more diverse population of interns, we conducted a nationwide study of US interns in which we collected monthly data on self-reported attentional failures (defined as “nodding off or falling asleep” during patient-care or educational activities), significant medical errors, and preventable adverse events (including fatalities).

## Methods

Details regarding participant recruitment have been described in detail elsewhere [4]. Briefly, in the spring of 2002, we sent email announcements to all individuals who successfully matched to a residency program in the National Residency Matching Program (NRMP) and to all known email addresses of graduating fourth-year medical students of US programs in an effort to reach as many of the 18,447 individuals who matched to residencies via the NRMP. These emails directed potential participants to a secure Web site which provided detailed information about the study and enabled participants to provide electronic informed consent.

In June 2002, an email was sent to the 3,429 interns who had volunteered to participate in the study, and they were directed to a password-coded secure Web site to complete a baseline survey that solicited background data. From July 2002 to May 2003, on the 28th day of each month, emails were sent to those who had completed the baseline survey (the study cohort) to direct participants to a secure Web site to complete a monthly survey. Monthly surveys were available on the Web site until the 27th day of the next month, and we ensured that each participant answered each monthly survey only once.

The monthly survey contained detailed questions regarding work hours, sleep, activities during the month, number of days off, and the number of extended-duration work shifts. Among the 60 questions they were asked each month, interns were asked to report whether they had made a significant medical error (“Do you believe sleep deprivation or fatigue caused you to make a significant medical error?” [henceforth referred to here as “fatigue-related errors”] and “Do you believe you made any significant medical errors other than due to sleep deprivation or fatigue?” [henceforth referred to here as “non-fatigue-related errors”]). If they answered affirmatively, they were directed to a supplementary survey that elicited further information about the error(s), including whether they had resulted in an adverse patient outcome (i.e., a preventable adverse event) or a patient fatality (i.e., a preventable adverse event resulting in a fatality). Moreover, interns were also asked to report how many times they had nodded off or had fallen asleep (attentional failure) during specific patient-care activities (during surgery and while talking to, or examining, patients) and educational activities (during rounds with the attending physicians and during lectures, seminars, or grand rounds). The remaining questions addressed secondary outcomes such as caffeine usage, health, and mood, and served as distracters for the main hypothesis.

The Web sites were hosted and maintained by Pearson Assessments (http://www.pearsonncs.com). Data were transmitted electronically on a weekly basis through secure means from Pearson Assessments to the Brigham and Women's Hospital. All demographic and potentially identifiable data were then stored separately from the main database. A certificate of confidentiality was issued by the Centers for Disease Control; the data were also protected by federal statute (Public Health Service Act 42 USC) as a consequence of funding from the Agency for Healthcare Research and Quality. The Brigham and Women's Hospital/Partners HealthCare System Human Research Committee approved the procedures for the protocol, and electronic informed consent was obtained from all participants.

### Work-Hour Validation

A random subset of participants (7%) completed daily work/sleep diaries, and these diaries were validated in a separate study using continuous work-hour monitoring by direct observation and polysomnographic recordings [2]. Those completing the work/sleep diaries recorded daily work hours for at least 21 out of 28 d and completed the corresponding monthly survey. Pearson product–moment correlation was used to determine the association between daily average work hours and number of extended-duration work shifts reported in the diary and through the monthly survey.

### Statistical Analysis

Our analysis to determine whether the number of extended-duration shifts worked per month was associated with significant medical errors and with attentional failures during surgery, while examining patients, while on rounds with attending physicians, and during educational events employed a case-crossover component of self-matching [5,6]. Specifically, months were classified according to the number of reported extended-duration shifts worked (i.e., zero, between one and four, and five or more shifts) per month and whether or not a particular outcome occurred at least once during the month. The denominator for our events was thus the intern-month. That is, each participant was considered as a separate stratum in the analysis, and therefore interns acted as their own controls. This case-crossover analysis thus eliminated the need to account for potential between-participant confounders such as age, gender, or medical specialty. A Mantel-Haenszel test was then used to calculate a pooled odds ratio of at least one outcome occurring during months with between one and four, or five or more extended-duration shifts worked (using months with no extended-duration shifts as the comparison group) [7].

To address potential reporting bias, we conducted a sub-analysis of the data from the 682 interns who completed all monthly surveys. In addition, given that the ACGME established new resident work-hour guidelines in 2003, we conducted a sub-analysis in which we included only those intern-months that were in compliance with current ACGME guidelines based on the frequency of extended-duration work shifts (i.e., those intern-months with nine or fewer extended-duration work shifts).

To limit this analysis to months in which interns worked full time, months reported to have fewer than 150 h worked were excluded. Odds ratios are reported with 95% confidence intervals [CIs]. SAS 8.2 (SAS Institute [http://www.sas.com]) was used for statistical analysis, and *p* < 0.05 defined statistical significance.

## Results

As we have reported elsewhere [4], 18.6% of the interns who were sent an advertisement announcing the study volunteered to participate and enrolled electronically in the study (3,429 interns). Of those volunteers, 2,737 completed the baseline survey and were considered to be the study cohort (80% response rate of volunteers). The mean number of surveys completed per participant was 7.2 ± 4.0 (mean ± standard deviation); 682 participants (25%) completed all 12 surveys. The number of surveys received per month ranged from 1,167 to 2,118 (1,548 ± 376). A total of 19,740 surveys (2,737 baseline surveys and 17,003 monthly surveys) were collected. Of the intern-months, 81% were in compliance with current ACGME guidelines with respect to the frequency of extended-duration work shifts.

Our survey cohort was 53% female and 47% male with a mean age of 28.0 ± 3.9 y. Compared with data obtained from the Electronic Residency Application Service (41% female, 56% male, mean age = 30.2 y [P. Jolly, Association of American Medical Colleges, personal communication]), our cohort was slightly younger and had a relatively greater proportion of women. Of our participants, 85% had graduated from US medical schools in 43 states, and 15% had graduated from foreign medical schools. Thus, we had fewer foreign medical graduates compared with the overall statistics from the NRMP (74% US medical graduates, 26% foreign medical graduates) [8]. The distribution of medical/surgical specialties of the survey participants was similar to the distribution of specialties matched through the NRMP [4]. The mean number of extended-duration work shifts reported per month was 3.9 ± 3.4 (median = 4; range = 0–18), and their average duration was 32.0 ± 3.7 h [4]. The mean weekly work hours reported in each of the three categories of extended-duration shifts per month (zero, between one and four, and five or more extended-duration shifts) were 55.5 ± 13.8 (29% of intern-months), 64.8 ± 15.5 (24% of intern-months), and 79.3 ± 14.8 h (47% of intern-months), respectively.

### Work-Hour Validation

As reported elsewhere [4], the Pearson correlation coefficients for the work hours and number of extended-duration work shifts reported in the monthly survey versus actual hours and extended-duration shifts worked, as indicated by the daily work diaries completed by a subset of our cohort, was *r* = 0.76 (*n* = 192, *p* < 0.001), and *r* = 0.94 (*n* = 40, *p* < 0.001), respectively.

### Significant Medical Errors and Preventable Adverse Events

The frequency of extended-duration work shifts had a substantial impact on reported rates of fatigue-related significant medical errors. That is, the reported rates of fatigue-related significant medical errors were increased substantially as the number of extended-duration work shifts increased (Table 1). Interns reported at least one fatigue-related significant medical error in 3.8% of months with no extended-duration shifts. However, at least one fatigue-related significant medical error was reported in 9.8% of months with between one and four extended-duration work shifts and 16% of months with five or more extended-duration work shifts. Reported rates of fatigue-related significant medical errors that resulted in a preventable adverse patient outcome also increased as the number of reported extended-duration shifts worked per month increased (0.2%, 1.1%, and 1.6%, respectively, in months with zero, between one and four, and five or more extended-duration shifts per month). A similar significant relationship was seen with fatigue-related errors associated with a fatality.

**Table 1:**
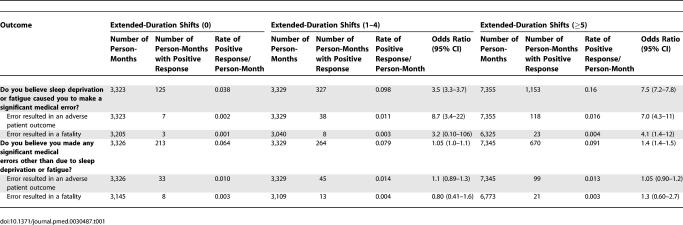
Self-Reported Significant Medical Errors, Adverse Events, and Fatal Errors per Person-Month, According to Sleep Deprivation or Fatigue, and Extended-Duration Shifts, with Corresponding Odds Ratios (95% CIs)

The odds ratios of reporting at least one significant medical error due to fatigue were 3.5 (95% CI, 3.3–3.7) and 7.5 (95% CI, 7.2–7.8), respectively, in months with between one and four reported extended-duration shifts and with five or more reported extended-duration shifts compared to months with no extended-duration shifts (reference group) (Table 1). A similar relationship was seen with fatigue-related preventable adverse events, with odds ratios of 8.7 (95% CI, 3.4–22) and 7.0 (95% CI, 4.3–11), respectively. Although the total number of medical errors resulting in a fatality was low, this rate also significantly increased in months with five or more extended-duration shifts per month (odds ratio = 4.1, 95% CI, 1.4–12). The number of reported fatigue-related medical errors increased as the number of extended-duration shifts per month increased regardless of gender or age. Males: odds ratio = 4.7 (95% CI, 4.0–5.4) and odds ratio = 8.8 (95% CI, 8.0–9.6) for between one and four extended-duration shifts and five or more extended-duration shifts per month, respectively. Females: odds ratio = 2.9 (95% CI, 2.6–3.2) and odds ratio = 6.7 (95% CI, 6.2–7.1) for between one and four extended-duration shifts and five or more extended-duration shifts per month, respectively. For participants below age 28 years, odds ratio = 3.8 (95% CI, 3.4–4.3) and odds ratio = 6.6 (95% CI, 6.1–7.2) for between one and four extended-duration shifts and five or more extended-duration shifts per month, respectively. For participants over age 28 years, odds ratio = 3.2 (95% CI, 2.8–3.7) and odds ratio = 8.6 (95% CI, 7.8–9.4) for between one and four extended-duration shifts and five or more extended-duration shifts per month, respectively (not shown in Table 1).

Significant medical errors made for reasons other than fatigue or sleep deprivation were not as strongly associated with the number of extended-duration shifts worked per month. Although rates of non-fatigue-related significant medical errors were greater in months with more frequent extended-duration work shifts, the relationship was modest (Table 1). Compared to months with no extended-duration work shifts, odds ratios of reporting at least one non-fatigue-related significant medical error were 1.05 (95% CI, 1.01–1.1) and 1.4 (95% CI, 1.4–1.5) during months with between one and four extended-duration shifts and with five or more extended-duration shifts, respectively. Rates of non-fatigue-related preventable adverse events were not significantly associated with the frequency of extended-duration work shifts. Compared to months with no extended-duration work shifts, the odds ratios of non-fatigue-related preventable adverse events were 1.1 (95% CI, 0.89–1.3) and 1.05 (95% CI, 0.9–1.2) in months with between one and four extended-duration shifts and with five or more extended-duration shifts, respectively.

To address the issue of reporting bias, we repeated the analysis of fatigue-related errors in only those participants who returned all surveys. In this subgroup (*n* = 682), the odds ratios of a fatigue-related significant medical error during months with between one and four extended-duration shifts and with five or more extended-duration shifts per month compared to months with no extended-duration shifts were similar to the analysis with all participants (odds ratio = 4.0 [95% CI, 3.5–4.4] and odds ratio = 6.7 [95% CI, 6.2–7.2], respectively) (not shown in Table 1). Odds ratios for fatigue-related preventable adverse events were similarly increased (odds ratio = 7.4 [95% CI, 1.9–28] and odds ratio = 4.4 [95% CI, 2.2–8.7], respectively, during months with between one and four extended-duration shifts and with five or more extended-duration shifts per month compared to months with no extended-duration shifts) (not shown in Table 1). Additional secondary analysis in which we limited the data to only those months on hospital wards yielded associations between the frequency of extended-duration work shifts and medical errors and adverse events that were comparable to the results of the overall analysis. Odds ratios for fatigue-related medical errors and preventable adverse events were 3.5 (95% CI, 3.3–3.7) and 7.5 (95% CI, 7.2–7.8), respectively (not shown in Table 1).

Results from analysis limited to interns in compliance with current ACGME guidelines with regard to frequency of extended-duration work shifts were comparable to those from the overall sample. In months with between five and nine extended-duration work shifts compared to months without extended-duration work shifts, the odds ratio for reporting fatigue-related significant medical errors was 7.8 (95% CI, 7.5–8.1); the odds ratio for reporting a fatigue-related medical error that resulted in an adverse event was 8.1 (95% CI, 4.7–14); and the odds ratio for reporting a fatigue-related medical error that resulted in a fatality was 4.6 (95% CI, 1.3–16) (not shown in Table 1).

### Attentional Failures

For patient-care activities, the probability of reporting at least one attentional failure per month (i.e., falling asleep during surgery or while examining patients) was strongly associated with the frequency of extended-duration work shifts (Table 2). The odds ratios and 95% CI of attentional failures during surgery were significantly increased in months with between one and four extended-duration shifts (odds ratio = 2.1; 95% CI, 1.7–2.7) and five or more extended-duration shifts (odds ratio = 1.4; 95% CI, 1.3–1.6) compared to the reference group (no extended-duration shifts). Increases were also found for attentional failures during patient examinations (odds ratio = 1.5 [95% CI, 1.3–1.7] for between one and four extended-duration shifts and 2.1 [95% CI, 2.0–2.2] for five or more extended-duration shifts) compared to the reference group (no extended-duration shifts).

**Table 2:**
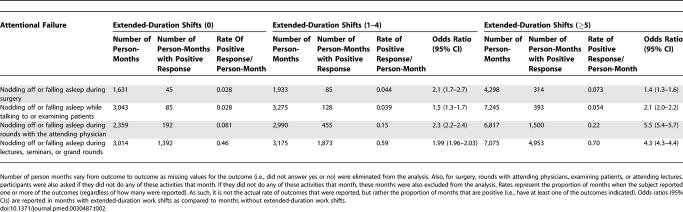
Self-Reported Attentional Failures per Person-Month with Corresponding Odds Ratios (95% CIs)

For educational activities (i.e., rounds with attending physicians or lectures), the results were more marked. The odds ratios of reporting at least one episode of falling asleep during rounds with attending physicians were significantly increased in months with between one and four extended-duration shifts (2.3; 95% CI, 2.2–2.4) and with five or more extended-duration shifts (5.5; 95% CI, 5.4–5.7). Odds ratios of falling asleep during lectures were similarly increased in months with between one and four extended-duration shifts (1.99; 95% CI, 1.96–2.03) and in months with five or more extended-duration shifts (4.3; 95% CI, 4.3–4.4), with 70% of intern-months with five or more extended-duration shifts having reports of falling asleep at least once during lectures. Interestingly, interns reported falling asleep during lectures at least once in 46% of months with no extended-duration work shifts.

### Sleep

Interns reported sleeping 179.3 ± 54.3 h (mean ± standard deviation) in months with no extended-duration work shifts, 176.6 ± 45.1 h in months with between one and four extended-duration work shifts, and 165.3 ± 39.2 h in months with five or more extended-duration work shifts.

## Discussion

We found that extended-duration work shifts were associated with adverse effects on patient safety (i.e., fatigue-related significant medical errors and preventable adverse events, including those resulting in fatalities). The risk of self-reported attentional failures during patient-care and educational activities was also significantly affected by extended-duration work shifts. That is, during months with frequent extended-duration work shifts (i.e., five or more extended-duration shifts in the month), interns were significantly more likely to fall asleep during surgery, while talking to or examining patients, during rounds, and during lectures or seminars, potentially affecting their ability to deliver patient care or to learn.

The hours of reported sleep per month decreased as the number of extended-duration work shifts, the number of reported medical errors, and the number of reported attentional failures increased—and this is consistent with laboratory studies that have unequivocally demonstrated the profound effects of sleep deprivation on alertness and performance [9–15]. Indeed, the adverse effects after 24 h of continuous wakefulness on neurobehavioral performance are comparable to that of a blood alcohol concentration of 0.10% [16,17]. Our results confirm the recently published findings by Landrigan et al. [3] and by Lockley et al. [2]. During a traditional schedule of extended-duration work shifts every other shift, as compared to a timetable where the scheduled length of work shifts was limited to less than 16 h, Landrigan et al. reported that serious medical errors were 36% more frequent [3], while Lockley et al. reported that polysomnographically defined attentional failures were approximately twice as frequent [2]. Moreover, our findings extend these results by confirming the associations between extended-duration work shifts, medical errors, preventable adverse events (including those resulting in fatalities), and attentional failures in interns in multiple specialties and hospitals across the United States.

In addition to the impact of these fatigue-related serious medical errors on patient safety, the perception of having made a medical error that causes an adverse patient outcome creates significant emotional distress for physicians that can last days or years and may include feelings of fear, guilt, anger, embarrassment, and humiliation [18]. The personal distress and reduced empathy associated with perceived medical errors increase the odds of future medical errors [19]. Without coping mechanisms, such as accepting responsibility, discussion with colleagues, and disclosure to patients, physicians may use dysfunctional methods of dealing with errors, such as alcohol and drug abuse [20,21].

We also demonstrated that extended-duration work shifts adversely affected medical education, as the odds of falling asleep in lectures and while on rounds with attending physicians increased significantly when extended-duration shifts were worked. Not only is adequate sleep crucial for maintaining vigilance [22], but sleep also plays an important role in memory consolidation and learning [23,24]. The amount of sleep reported by most interns is less than that shown to be necessary to perform satisfactorily on cognitive performance tasks that are implicitly necessary for learning [25]. Additionally, the high level of chronic partial sleep deprivation, consistent with prior reports [26,27], may account for the high base rate of attentional failures in these participants.

We acknowledge that, despite the large amount of data on intern-months collected from interns across specialties from programs throughout the United States, there are a number of limitations to our study. First, the individuals who volunteered for our study represent a small proportion of interns in the United States. Therefore, it is possible that the participants in our study may not be representative of the entire population of interns. For instance, our sample could be biased towards interns with a specific interest in the issue of resident work hours, thus raising the potential of a reporting bias. Additionally, those individuals who responded to our survey may have been more responsible and therefore more likely to report medical errors (although the responsibility of the participant could not account for the relationship between the numbers of extended-duration shifts and reported medical errors that were identified by the within-person analysis). However, potential participants were not informed of the specific study hypotheses, and the questions addressing our primary exposure and outcome variables were distributed among questions regarding secondary outcome measures such as caffeine usage, mood, and stress. Moreover, because each intern serves as his/her own control in our case-crossover analysis, it would have taken considerable time over a number of months—and a conscious effort to mislead—for intentionally biased respondents to affect our results deliberately. However, it remains a possibility that those who were more attentive to the topic of medical errors may have volunteered to participate in the study.

Second, the data for medical errors relied on self-reports and were not independently validated. Additionally, because “fatigue or sleep deprivation” was part of the question regarding medical-error outcomes, it may have inflated the association between this suggested “cause” and the medical error. Third, the definition of what incidents interns believe constituted a ”significant error” or “adverse patient outcome” may have varied from person to person. However, the consistency of the results when the outcome of the error resulted in a fatality provides a common metric of severity. In addition, because each intern was compared only to him- or herself, there is no reason to believe that he or she would have changed the criteria for what was significant by the nature of the rotation schedule.

Fourth, in our case-crossover analysis, it is possible that during months without extended-duration work shifts, interns may be on rotations with fewer opportunities to make errors (e.g., radiology rotation, dermatology, ambulatory elective), than on more clinically-oriented rotations when extended-duration work-shift frequency was greater (e.g., intensive-care unit, hospital ward) resulting in more opportunities to make errors. Additionally, there may be other additional organizational features (e.g., staffing, supervision) that differ between rotations. To address partially this issue of monthly activity, we conducted a secondary analysis in which we limited the data to only those months on hospital wards. When we did so, we found that the association between the frequency of extended-duration work shifts and medical errors and adverse events was comparable to the results of the overall analysis. While the number of hours at work increases as the number of shifts increases and may partially account for the increased rate of events, it should be noted that the percentage increase in weekly work hours (a 16.7% increase in the category of between one and four extended-duration shifts per month and a 42.9% increase in the number of five or more extended-duration shifts per month, compared to months with no extended-duration shifts) cannot account for the observed increase in the rate of events. For months with between one and four extended-duration shifts and for months with five or more extended-duration shifts, fatigue-related adverse events increased by ∼450% and ∼700%, respectively. For months with five or more extended-duration shifts, fatigue-related adverse events resulting in a fatality increased by ∼300%.

We also recorded the frequency of both fatigue-related and non-fatigue-related errors. We found that even though the rate of non-fatigue-related errors increased as extended-duration work-shift frequency increased (presumably for the two reasons outlined above), the magnitude of the increase was much less than the increased rate of fatigue-related errors. This finding suggests that the increased rate of fatigue-related errors was not predominately due to a confounding effect of monthly activity. However, it remains possible that interns were more tired when working more frequent extended-duration shifts and that they may have been more likely to under-report or to over-report medical errors, misperceive errors in their work, or to attribute errors to fatigue. Landrigan and colleagues found that physicians perceived fewer medical errors than those assessed by an independent observer [3].

Fifth, for many of our analyses, we collected information about the events (i.e., errors) and exposures (i.e., number of extended-duration shifts worked per month) simultaneously from the monthly surveys. This could lead to reporting bias as interns may be more inclined to send back surveys during months in which incidents occurred. However, when analyses were limited to participants returning all surveys throughout the year, the relationships between errors and the frequency of extended-duration shifts did not change substantially. Sixth, there may be a carry-over effect from one month to the next. For example, an intern may have worked five extended-duration shifts in the first month and no extended-duration shifts in the second month. If that intern reported a medical error at the beginning of the second month, our data analysis would not have been able to detect the potential carry-over effect from the high number of extended-duration shifts in the first month.

Finally, although self-reporting is a well-established method of eliciting information regarding medical errors, prior work has demonstrated that even very robust stimulated self-reporting systems detect only a fraction of all medical errors [28]. Consequently, it is likely that the interns committed a greater number of serious medical errors, including those resulting in preventable adverse events, than we have reported here. Thus, although there are a number of potential biases and confounders in our study, given the consistency and magnitude of our findings, it is unlikely that they could account for the striking association between extended-duration work shifts and fatigue-related significant medical errors, preventable adverse events, and attentional failures.

According to the 1999 report from the Institute of Medicine, between 48,000 and 98,000 deaths each year occur due to a medical error [1]. Recently, the ACGME has placed limitations on resident work hours in an attempt to reduce fatigue-related medical errors. Historically, when such attempts have been made to reduce resident work hours in the United States, the frequency of extended-duration work shifts (>24 h) has been reduced (e.g., from a frequency of Q2 to Q3 to Q4 [where Q2 is an extended duration shift occurring every other night, Q3 is an extended duration shift occurring every third night, and Q4 is an extended duration shift occurring every fourth night]). However, the practice of working for more than 24 h consecutively has remained the cornerstone of American postgraduate medical education. In fact, the recent (2003) ACGME work-hour guidelines for postgraduate medical education programs effectively continue to sanction up to nine extended-duration shifts (of up to 30 h consecutively) per month, since every other shift can be an extended-duration work shift under the new ACGME guidelines [29]. Still, interns working extended-duration shifts within these ACGME guidelines reported significant numbers of medical errors, including those that resulted in adverse patient outcomes and fatalities. Furthermore, 83.6% of interns reported working more hours than allowed by ACGME standards in the year following their introduction [30].

These data, collected from interns in all specialties across the United States, are not consistent with the recent suggestions by a member of the ACGME Residency Review Committee in Surgery [31] that safety hazards associated with resident fatigue are limited to a small subset of trainees. Even interns who worked well below the current 80-h ACGME weekly work-hour limits (averaging 64.8 h of work per week), but who continued to work up to one extended-duration shift per week (half the weekly frequency allowed under current ACGME standards), had 8-fold greater odds of reporting an adverse event than those who did not work extended-duration work shifts. This finding is consistent with data from numerous studies documenting that 24 h consecutively of wakefulness impairs short-term memory, degrades neurobehavioral performance, and greatly increases the risk of both errors of commission and omission and attentional failures [23,32,33]. Additionally, Ayas and colleagues recently reported that the odds of an intern having a percutaneous injury increased by 61% after ≥ 20 h at work [34]. These findings are also consistent with the recent demonstration that elimination of extended-duration work shifts reduces attentional failures and serious medical errors among interns working in intensive-care units [2,3].

Our results thus reveal that the practice of scheduling 24-h or greater extended-duration work shifts, as currently sanctioned by the ACGME, may pose a significant increased risk of safety hazards to patients, contribute to the occurrence of medical errors that are attributable to fatigue or sleep deprivation and to consequent preventable fatal and nonfatal adverse events, and may also interfere with the primary educational purpose of residency training. These results have important public policy implications in terms of postgraduate medical education and suggest that directors of training programs should consider alternative coverage schedules for trainees with the objective of eliminating extended-duration shifts. In Europe, where the tradition of extended-duration “on call” shifts originated more than a century ago, work shifts of all physicians (including those in training) have recently been limited to 13 h consecutively [35], thereby eliminating extended-duration work shifts altogether. Fletcher et al. recently published a review of interventions aimed at reducing US resident work hours, including strategies such as day and night float teams and the use of physician extenders [36]. Future studies should explore the applicability of our findings regarding the association between medical errors and extended-duration work shifts to all practicing physicians in the United States.

## Supporting Information

Alternative Language Abstract S1 Translation of the Abstract into Hungarian by S. Kantor (102 KB PDF)

Alternative Language Abstract S2 Translation of the Abstract into Polish by L. Kubin (27 KB PDF)

Alternative Language Abstract S3 Translation of the Abstract into Portuguese by F. Louzada (22 KB PDF)

Alternative Language Abstract S4 Translation of the Abstract into French by C. Gronfier (32 KB PDF)

Alternative Language Abstract S5 Translation of the Abstract into Chinese by L. Ling (188 KB PDF)

Alternative Language Abstract S6 Translation of the Abstract into Spanish by C. Robles (28 KB PDF)

Alternative Language Abstract S7 Translation of the Abstract into Japanese by T. Tanigawa (16.5 KB PDF)

## Abbreviations

ACGME: Accreditation Council for Graduate Medical Education
AHRQ: Agency for Healthcare Research and Quality
CI: confidence interval
NRMP: National Residency Matching Program
