# Supplementary material for: Impact of Extended-Duration Shifts on Medical Errors, Adverse Events, and Attentional Failures
Source: PLoS Med. 2006 Dec 12;3(12):e487. doi: 10.1371/journal.pmed.0030487 (PMC1705824; doi:10.1371/journal.pmed.0030487)
Supplement: Supplementary file 1 [file pmed.0030487.sd001.pdf]

A hosszú műszakok hátasa az orvosi műhibákra, szövődményekre és figyelemhiányra

Előzmények: Egy, a közelmúltban végzett, kontrollalt vizsgálatban kimutatták, hogy súlyos betegeket ellátó intézményekben a hosszú (24 órás vagy annál is hosszabb) műszakok megszüntetése csökkenti a jelentős orvosi műhibák, és a poliszomnográfiával vizsgált figyelemhiány arányát. Az eredmények felvetették annak lehetőségét, hogy a rezidens orvosoknál rendszeresen előforduló hosszú műszakok általánosabban fokozzák az orvosi műhibák és talán a szövődmények veszélyét. A jelenlegi vizsgálatot Amerika-szerte, rezidens orvosok változatos populációján végeztük el arra keresve a választ, hogy vajon a hosszú műszakok rezidenseknél összefüggnek-e a súlyosabb orvosi műhibákkal, szövődményekkel és figyelemhiánnyal.

Módszerek: Az általunk végzett prospektív, országos, internetes felmerés során 2737 elsőéves rezidens 17003 havi jelentést töltött ki. Minden hónapban, a hosszú műszakok száma és a jelentős orvosi hibákat, elkerülhető szövődményeket és figyelemzavart tartalmazó beszámolók közötti kapcsolatot un. „case-crossover" modszerrel vizsgáltuk, amely során minden kísérleti alany egyben önmaga kontrollja is volt.

Eredmények: A hosszú műszak-mentes hónapokhoz viszonyítva, azokban a hónapokban, amelyekben 1-4 illetve 5 vagy annál is több hosszú műszak volt, a valószínűségi rátája annak, hogy legalább egy, fáradtság okozta, jelentős orvosi műhiba bekövetkezik 3,5 (95% konfidencia intervallum: 3,3-3,7) illetve 7,5 (7,2-7,8). A kimerültség okozta szövődmények valószínűségi rátája, amely egyébkent 8,7 (3,4-22) és 7,0 (4,3-11) volt, szinten növekedett. Azok a rezidensek, akik ötnél több hosszú műszakot vállaltak havonta, gyakrabban számoltak be figyelemzavarról, amely az előadások, megbeszélések, és klinikai tevékenységük során jelentkezett beleértve a műtéteket is.

Az eredmények értelmezése: A vizsgált rezidenseknél országszerte a hosszú műszakok kapcsolatban álltak a súlyos orvosi műhibák, a szövődmények és a figyelemzavar fokozott veszélyével. Az eredményeknek jelentős, közérdeket is érintő hatása van a posztgraduális orvos képzésre.
