# Supplementary material for: Impact of Extended-Duration Shifts on Medical Errors, Adverse Events, and Attentional Failures
Source: PLoS Med. 2006 Dec 12;3(12):e487. doi: 10.1371/journal.pmed.0030487 (PMC1705824; doi:10.1371/journal.pmed.0030487)
Supplement: Supplementary file 2 [file pmed.0030487.sd005.pdf]

长时轮班工作对医疗错误, 不良事件及注意力不集中的影响

引言：最近, 在重症监护病房中采用随机抽样的一项研究显示, 消除长时间的轮班工作(◎24小时)可降低主要医疗错误以及由多导睡眠图仪记录的注意力不集中的发生率。这项结果引起了人们对实习医生中普遍存在的长时轮班工作的关注, 因为长时轮班工作或许会更广泛地增加发生医疗错误与医疗不良事件的危险。本项研究的目的是评估在美国实习医生这样一个多样性群体中, 长时轮班工作同主要医疗错误, 不良事件及注意力不集中之间是否存在相关关系。

方法：我们在全美国通过互联网进行了一次调查, 其中共有 2,737 名医学院毕业后第一年的实习医生接受了调查并填写了 17,003 个月度报告。我们用病例-交叉设计的方法, 以每个实习医生作为他(她)自己的正常对照, 分析了一个月内长时轮班工作的次数同主要医疗错误, 可预防的不良事件及注意力不集中的出现次数之间的相关性。

结果：同没有长时轮班的月份相比, 在含有1-4次以及含有◎5次长时轮班的月份中, 发生至少一次与疲劳有关的主要医疗错误的机会比率分别是3.5(95%置信区间为3.3-3.7)和7.5(7.2-7.8)。与疲劳有关的医疗不良事件发生的机会比率也增加了, 分别为8.7(3.4-22)和7.0(4.3-11)。另外, 在长时轮班多于5次的月份里, 那些实习医生听课和研讨会以及进行临床活动(包括手术)时注意力不集中的次数也增多了。

结论：在全美国范围的实习医生中, 长时轮班工作同出现主要医疗错误, 不良事件及注意力不集中的增加有关联。这些结果对医学院毕业后医学教育的公共政策具有重要意义。
