# Supplementary material for: Impact of Extended-Duration Shifts on Medical Errors, Adverse Events, and Attentional Failures
Source: PLoS Med. 2006 Dec 12;3(12):e487. doi: 10.1371/journal.pmed.0030487 (PMC1705824; doi:10.1371/journal.pmed.0030487)
Supplement: Supplementary file 3 [file pmed.0030487.sd007.pdf]

長時間勤務が医療過誤、患者への有害事象および注意不全に及ぼす影響

背景：救急病棟における最近の無作為対照試験によって、２４時間以上の超過勤務をなくすことで重大な医療過誤や終夜睡眠ポリグラフ検査によって記録される注意不全の割合が減ることが明らかにされた。これは、インターンで定常化している超過勤務が、従来考えられているよりもさらに一般的に医療過誤や、おそらくは患者への有害事象のリスクにつながるかもしれないという懸念を生じさせた。本研究では全米の多様なインターン集団において、超過勤務労働と重大な医療過誤、患者への有害事象、注意不全などとの関連を明らかにした。
方法：われわれは、全米 Web による前向き調査を実施した。２７３７名の卒後１年目の研修医（インターン）が１７００３件の月間報告を完了した。月毎の超過間勤務の回数と重大な医療過誤の報告、予防可能な患者への有害事象、注意不全との関連について各インターン自身を対照とした症例クロスオーバー分析を行った。
知見：超過勤務がなかった月と比べて、１－４時間および 5 時間以上の超過勤務があった月においては、少なくとも１件以上の疲労関連の重大な医療過誤に関する報告数のオッズ比は、それぞれ 3.5（95%信頼区間 3.3-3.7）と 7.5（同 7.2-7.8）であった。疲労と関連した患者への有蓋事象のオッズ比も、それぞれ 8.7（同 3.4-22）と 7.0（同 4.3-11）と増加していた。月 5 回以上の超過勤務をしたインターンは講義中、回診中および手術を含む臨床活動中に、より多くの注意不全を報告した。
解釈：全米のインターン集団において超過勤務は、重大な医療過誤や患者への有害事象および注意不全などのリスクの増加と関連していた。これらの結果は、卒後臨床研修医教育について重要な公的施策上の示唆に富む。

Takeshi Tanigawa, MD
University of Tsukuba, JAPAN
