# Supplementary material for: Impact of Extended-Duration Shifts on Medical Errors, Adverse Events, and Attentional Failures
Source: PLoS Med. 2006 Dec 12;3(12):e487. doi: 10.1371/journal.pmed.0030487 (PMC1705824; doi:10.1371/journal.pmed.0030487)
Supplement: Supplementary file 4 [file pmed.0030487.sd002.doc]

**Wpływ długich dyżurów na błędy lekarskie, wypadki medyczne i zaburzenia koncentracji**

*Wstęp:* Badania statystyczne przeprowadzone wśród personelu oddziałów intensywnej terapii wykazały ostatnio, że zniesienie długich dyżurów (ponad 24 godz.) zmniejsza częstość występowania poważnych błędów lekarskich oraz poprawia mierzoną metodami poligraficznymi zdolność do koncentracji. Wyniki te zasugerowały, że powszechna wsród stażystów praca na długich dyżurach może zwiększać ryzyko błędów i wypadków w opiece lekarskiej. Celem obecnych badań było określenie czy praca na długich dyżurach jest związana z podwyższoną częstością błedów, wypadków i zaburzeń koncentracji u stażystów pracujących w rożnych ośrodkach służby zdrowia w Stanach Zjednoczonych.

*Metody:* Prospektywnymi, ogólnokrajowymi badaniami objęto 2737 osób odbywających pierwszy rok stażu po ukończeniu studiów medycznych, które ogółem wypełniły w miesięcznych odstępach 17.003 elektronicznych kwestionariuszy. Dla każdego ankietowanego obliczono poziom korelacji pomiędzy miesięczną liczbą długich dyżurów a ilością stwierdzonych poważnych błędów lekarskich, zapobiegalnych wypadków oraz przypadków zaburzeń uwagi.

*Wyniki:* W porównaniu z miesiącami bez pracy na długich dyżurach, prawdopodobieństwo wystąpienia przynajmniej jednego poważnego błędu lekarskiego w miesiącu, w którym badani pracowali na 1-4 długich dyżurach było podwyższone 3,5-krotnie (95% zakres ufności: 3,3-3,7), a w miesiącach z 5 lub wiekszą liczbą długich dyżurów, 7,5-krotnie (7,2-7,8). Prawdopodobieństwa występowania wypadków medycznych związanych ze zmęczeniem były odpowiednio podwyższone 8,7-krotnie (3,4-22) i 7-krotnie (4,3-11). Stażyści pracujący na ponad 5 długich dyżurach miesięcznie stwierdzali podwyższoną czestość zaburzen koncentracji w czasie wykładów, obchodów, oraz w pracy klinicznej, w tym w czasie zabiegów chirurgicznych.

*Interpretacja:* Praca na długich dyżurach zwiazana jest z podwyższonym ryzykiem poważnych błędów lekarskich, wypadków oraz zaburzeń uwagi u lekarzy-stażystów. Wyniki te zawierają ważne przesłanki dla reformy podyplomowego kształcenia lekarzy.
