# Supplementary material for: Impact of Extended-Duration Shifts on Medical Errors, Adverse Events, and Attentional Failures
Source: PLoS Med. 2006 Dec 12;3(12):e487. doi: 10.1371/journal.pmed.0030487 (PMC1705824; doi:10.1371/journal.pmed.0030487)
Supplement: Supplementary file 5 [file pmed.0030487.sd003.doc]

**Impacto das Longas Jornadas sobre os Erros Médicos, Eventos Adversos e Falhas de Atenção**

*Histórico*: Um ensaio controlado e randomizado realizado recentemente em unidades críticas de tratamento mostrou que a eliminação de longas jornadas de trabalho (≥24 horas) reduz as taxas de erros médicos significativos e falhas de atenção registradas através de polissonografia. Deste resultado originou a preocupação de que estas longas jornadas de trabalho, comumente observadas no trabalho de médicos residentes, pode contribuir para o risco de erros médicos e talvez para a ocorrência de eventos adversos de maneira geral. Este estudo avaliou se as longas jornadas de trabalho estão associadas a erros médicos significativos, eventos adversos e falhas de atenção em uma população de médicos residentes nos EUA.

*Metodologia*: Nόs realizamos uma pesquisa prospectiva, baseada em informações obtidas em levantamento feito via Web, na qual 2737 residentes em seu primeiro ano de residência completaram 17,003 relatos mensais. A associação entre o número de longas jornadas trabalhadas por mês e o número de erros médicos significativos, eventos adversos que poderiam ser evitados e falhas de atenção foi obtida através da utilização de uma análise sequencial, na qual cada sujeito é controle dele mesmo.

*Resultados*: Na comparação entre meses nos quais não ocorreram longas jornadas de trabalho e meses nos quais ocorreram entre 1-4 longas jornadas e ≥5 longas jornadas, as odds ratios relativas ao relato de ao menos um erro médico significativo relacionado à fadiga foram 3.5 (95%CI: 3.3-3.7), e 7.5 (7.2-7.8), respectivamente. As respectivas odds ratios para eventos adversos relacionados à fadiga, 8.7, (3.4-22) e 7.0 (4.3-11), também apresentaram aumento. Residentes que trabalharam mais de 5 longas jornadas por mês relataram mais falhas de atenção durante palestras, reuniões e atividades clínicas, incluindo cirurgias.

*Interpretação*: Longas jornadas de trabalho mostraram associação com um risco aumentado de erros médicos significativos, eventos adversos e falhas de atenção em residentes norte-americanos. Os resultados têm importantes implicacões para a definicão de políticas públicas relacionadas à formacão médica.
