# Supplementary material for: Impact of Extended-Duration Shifts on Medical Errors, Adverse Events, and Attentional Failures
Source: PLoS Med. 2006 Dec 12;3(12):e487. doi: 10.1371/journal.pmed.0030487 (PMC1705824; doi:10.1371/journal.pmed.0030487)
Supplement: Supplementary file 6 [file pmed.0030487.sd004.doc]

**Impact des gardes de longue durée sur les erreurs médicales, les événements négatifs et les déficits attentionnels**

*Introduction* : Une récente étude randomisée, effectuée dans des unités de soins intensifs, a révélé que la suppression des gardes de longue durée (≥24 heures) réduit le taux d’erreurs médicales significatives et des déficits attentionnels enregistrés par polysomnographie. Ceci a suscité l’inquiétude que les gardes de longue durée, communément effectuées par les internes, pourraient contribuer au risque d’erreurs médicales et peut être à des événements négatifs. La présente étude à investigué dans quelle mesure les gardes de longue durée travaillés par les internes sont associées à des erreurs médicales significatives, des événements négatifs et des déficits attentionnels, dans une population variée d’internes sur l’ensemble des Etats-Unis.

*Méthodes* : Nous avons mené une enquête nationale prospective via internet pour laquelle 2373 internes (en 1ere année d’internat) ont complété 17003 rapports mensuels. L’association entre le nombre de gardes de longue durée travaillées dans le mois et le nombre déclaré d’erreurs médicales significatives, d’événements négatifs évitables et de déficits attentionnels ont été estimées en utilisant une analyse croisée individuelle dans laquelle chaque sujet a été utilisé comme son propre contrôle.

*Résultats* : Les odds-ratios de déclarer au moins une erreur médicale significative liée à la fatigue sont de 3.5 (95%CI: 3.3-3.7) pour les mois durant lesquels aucun poste de longue durée n’a été travaillé et de 7.5 (7.2-7.8) pour les mois durant lesquels 1-4 et ≥5 postes de longue durée ont été travaillés. Les odds-ratios pour les événements négatifs liés à la fatigue, respectivement de 7.0 (4.3-11) et 8.7, (3.4-22), sont aussi augmentés. Les internes effectuant plus de 5 gardes de longue durée par mois déclarent plus de déficits attentionnels durant les cours, les présentations de cas et les activités cliniques, incluant les opérations chirurgicales.

*Interprétation* : Les gardes de longue durée sont associées à un risque accru d’erreurs médicales significatives, d’événements négatifs, et de déficits attentionnels chez les internes au niveau national. Ces résultats ont des implications importantes dans la réglementation de la formation médicale.

**Impact of Extended Shifts on Medical Errors, Adverse Events and Attentional Failures**

**Impact des gardes de longue durée sur les erreurs médicales, les événements négatifs et les déficits attentionnels**

*Background*: A recent randomized controlled trial in critical care units revealed that the elimination of extended duration work shifts (≥24 hours) reduces the rates of significant medical errors and polysomnographically-recorded attentional failures.

Une récente étude randomisée, effectuée dans des unités de soins intensifs, a révélé que la suppression des gardes de longue durée (≥24 heures) réduit le taux d’erreurs médicales significatives et des déficits attentionnels enregistrés par polysomnographie.

This raised the concern that the extended duration shifts commonly worked by interns may contribute to the risk of medical errors, and perhaps adverse events more generally.

Ceci a suscité l’inquiétude que les gardes de longue durée, communément effectuées par les internes, pourraient contribuer au risque d’erreurs médicales et peut être à des événements négatifs.

This study assessed whether extended duration shifts worked by interns are associated with significant medical errors, adverse events and attentional failures in a diverse population of interns across the U.S.

La présente étude à investigué dans quelle mesure les gardes de longue durée travaillés par les internes sont associées à des erreurs médicales significatives, des événements négatifs et des déficits attentionnels, dans une population variée d’internes sur l’ensemble des Etats-Unis.

*Methods*: We conducted a prospective, nationwide Web-based survey in which 2737 residents in their first postgraduate year (interns) completed 17,003 monthly reports.

Nous avons mené une enquête nationale prospective via internet pour laquelle 2373 internes (en 1ere année d’internat) ont complété 17003 rapports mensuels.

The association between the number of extended duration shifts worked in the month and the reporting of significant medical errors, preventable adverse events, and attentional failures was assessed using a case-crossover analysis in which each subject acted as his/her own control.

L’association entre le nombre de gardes de longue durée travaillées dans le mois et le nombre déclaré d’erreurs médicales significatives, d’événements négatifs évitables et de déficits attentionnels ont été estimées en utilisant une analyse croisée individuelle dans laquelle chaque sujet a été utilisé comme son propre contrôle.

*Findings*: Compared to months in which no extended shifts were worked, during months in which 1-4 and ≥5 extended duration shifts were worked, the odds ratios of reporting at least one fatigue-related significant medical error were 3.5 (95%CI: 3.3-3.7), and 7.5 (7.2-7.8), respectively.

Les odds-ratios de déclarer au moins une erreur médicale significative liée à la fatigue sont de 3.5 (95%CI: 3.3-3.7) pour les mois durant lesquels aucun poste de longue durée n’a été travaillé et de 7.5 (7.2-7.8) pour les mois durant lesquels 1-4 et ≥5 postes de longue durée ont été travaillés.

The respective odds ratios for fatigue-related adverse events 8.7, (3.4-22) and 7.0 (4.3-11) were also increased.

Les odds-ratios pour les événements négatifs liés à la fatigue, respectivement de 7.0 (4.3-11) et 8.7, (3.4-22), sont aussi augmentés.

Interns working more than 5 extended duration shifts per month reported more attentional failures during lectures, during rounds, and during clinical activities, including surgery.

Les internes effectuant plus de 5 gardes de longue durée par mois déclarent plus de déficits attentionnels durant les cours, les présentations de cas et les activités cliniques, incluant les opérations chirurgicales.

*Interpretation*: Extended duration work shifts were associated with an increased risk of significant medical errors, adverse events, and attentional failures in interns nationwide. These results have important public policy implications for post-graduate medical education.

Les gardes de longue durée sont associées à un risque accru d’erreurs médicales significatives, d’événements négatifs, et de déficits attentionnels chez les internes au niveau national. Ces résultats ont des implications importantes dans la réglementation de la formation médicale.
