# Supplementary material for: Impact of Extended-Duration Shifts on Medical Errors, Adverse Events, and Attentional Failures
Source: PLoS Med. 2006 Dec 12;3(12):e487. doi: 10.1371/journal.pmed.0030487 (PMC1705824; doi:10.1371/journal.pmed.0030487)
Supplement: Supplementary file 7 [file pmed.0030487.sd006.doc]

**Impacto de turnos médicos prolongados en errores médicos, acontecimientos adversos y fallos de atención**

*Antecedentes:* Un reciente ensayo clínico controlado llevado a cabo en unidades de cuidados intensivos ha demostrado que la eliminación de los turnos prolongados de trabajo (≥24 horas) reduce el número de errores médicos y fallos de atención determinados por polisonografía. Esto ha incrementado la preocupación sobre la influencia que tienen los turnos médicos prolongados, trabajados normalmente por los interinos, en el riesgo de cometer errores médicos, y quizá de forma más general en otros acontecimientos adversos. El presente estudio evalúa si la duración prolongada de los turnos médicos esta asociada de forma significativa con errores médicos, con acontecimientos adversos y con fallos de atención, en diversos grupos de interinos en U.S.

*Métodos*: Se realizó una encuesta electrónica a nivel nacional en la cual 2737 residentes de primer año (interinos) completaron 17003 informes mensuales. Para el análisis, se usó el estudio cruzado de casos, en el cual el mismo sujeto actúa como su propio control. Con ello analizó la relación que existe entre el número de turnos prolongados trabajados en el mes y el número de errores médicos, acontecimientos adversos evitables y fallos de atención.

*Resultados*: La razón de probabilidad de cometer errores médicos, relacionados con la fatiga, durante los meses en los que los interinos hicieron de 1-4 y ≥5 turnos prolongados, en comparación con meses sin turnos largos, fue respectivamente (95%CI: 3.3-3.7) y 7.5 (7.2-7.8).

Asimismo, los acontecimientos adversos inducidos por la fatiga también se incrementaron, siendo la razón de probabilidad 8.7 (3.4-22) y 7.0 (4.3-11) respectivamente. Aquellos interinos que trabajaron más de 5 turnos prolongados por mes indicaron haber sufrido más fallos de atención durante conferencias, ronda de visitas y actividades clínicas, incluyendo cirugía.

*Conclusión*: La duración prolongada de los turnos de trabajo de los interinos a nivel nacional está asociada a un elevado riesgo de cometer importantes errores médicos, acontecimientos adversos y fallos de atención. Estos resultados tienen importante transcendencia pública para la educación de postgraduados de medicina.
